# Tafenoquine and NPC-1161B require CYP 2D metabolism for anti-malarial activity: implications for the 8-aminoquinoline class of anti-malarial compounds

**DOI:** 10.1186/1475-2875-13-2

**Published:** 2014-01-03

**Authors:** Sean R Marcsisin, Jason C Sousa, Gregory A Reichard, Diana Caridha, Qiang Zeng, Norma Roncal, Ronan McNulty, Julio Careagabarja, Richard J Sciotti, Jason W Bennett, Victor E Zottig, Gregory Deye, Qigui Li, Lisa Read, Mark Hickman, N P Dhammika Nanayakkara, Larry A Walker, Bryan Smith, Victor Melendez, Brandon S Pybus

**Affiliations:** 1 Division of Experimental Therapeutics, Military Malaria Research Program, Walter Reed Army Institute of Research, 503 Robert Grant Ave, Silver Spring, MD 20910, USA; 2 Malaria Vaccine Branch, Military Malaria Research Program, Walter Reed Army Institute of Research, Silver Spring, MD 20910, USA; 3 Center for Natural Products Research, University of Mississippi, Oxford, MS 38677, USA; 4 Center for Natural Products Research and Department of Pharmacology, School of Pharmacy, University of Mississippi, Oxford, MS 38677, USA; 5 United States Army Medical Material Development Activity, Frederick, MD 21701, USA

## Abstract

**Background:**

Tafenoquine (TQ) is an 8-aminoquinoline (8AQ) that has been tested in several Phase II and Phase III clinical studies and is currently in late stage development as an anti-malarial prophylactic agent. NPC-1161B is a promising 8AQ in late preclinical development. It has recently been reported that the 8AQ drug primaquine requires metabolic activation by CYP 2D6 for efficacy in humans and in mice, highlighting the importance of pharmacogenomics in the target population when administering primaquine. A logical follow-up study was to determine whether CYP 2D activation is required for other compounds in the 8AQ structural class.

**Methods:**

In the present study, the anti-malarial activities of NPC-1161B and TQ were assessed against luciferase expressing *Plasmodium berghei* in CYP 2D knock-out mice in comparison with normal C57BL/6 mice (WT) and with humanized/CYP 2D6 knock-in mice by monitoring luminescence with an *in vivo* imaging system. These experiments were designed to determine the direct effects of CYP 2D metabolic activation on the anti-malarial efficacy of NPC-1161B and TQ.

**Results:**

NPC-1161B and TQ exhibited no anti-malarial activity in CYP 2D knock-out mice when dosed at their ED_100_ values (1 mg/kg and 3 mg/kg, respectively) established in WT mice. TQ anti-malarial activity was partially restored in humanized/CYP 2D6 knock-in mice when tested at two times its ED_100_.

**Conclusions:**

The results reported here strongly suggest that metabolism of NPC-1161B and TQ by the CYP 2D enzyme class is essential for their anti-malarial activity. Furthermore, these results may provide a possible explanation for therapeutic failures for patients who do not respond to 8AQ treatment for relapsing malaria. Because CYP 2D6 is highly polymorphic, variable expression of this enzyme in humans represents a significant pharmacogenomic liability for 8AQs which require CYP 2D metabolic activation for efficacy, particularly for large-scale prophylaxis and eradication campaigns.

## Background

The 8-aminoquinoline (8AQ) class of anti-malarial compounds is extremely important in the fight against malaria, as this class of molecules is unique due to the efficacy against relapsing forms of malaria [1,2]. This activity is a result of the anti-hypnozoite activity of the 8AQ class [2,3]. This attribute along with gametocytocidal activity of primaquine (PQ) and other 8AQs make the class attractive for mass administration in efforts towards malaria eradication [4].

Recent advances in understanding the mechanism of action for the 8AQ drug PQ have been reported [5-7]. PQ is metabolized by several different CYP enzymes as well as monoamine oxidases [5-10], however, the anti-malarial activity of PQ is mediated through CYP 2D6-dependent activation to phenolic metabolites [6,7]. These metabolites are capable of redox cycling and generating reactive oxygen species [10-14], which is likely responsible for anti-malarial activity. The activity of PQ was shown to be dependent on CYP 2D6 activation in mouse models and a human clinical trial. The results reported by Pybus *et al.* clearly demonstrated in a mouse CYP 2D knock-out model that PQ was inactive against *Plasmodium berghei* and that the anti-malarial activity was partially restored at its ED_100_ dose in a humanized/CYP 2D6 knock-in mouse strain [6]. Bennett *et al.* demonstrated an association between poor and intermediate CYP 2D6 metabolizer phenotypes and failure of PQ for radical cure of *P. vivax* in two human subjects [15].

CYP 2D6 belongs to the cytochrome P450 super family of enzymes that are responsible for a variety of metabolic and biosynthetic processes [16]. Humans have several different hepatic CYP P450 enzymes that metabolize drugs to include CYP 1A1, 1A2, 2A6, 2B6, 2C19, 2C9, 2D6, 2E1, and 3A4. Each CYP isoform has different substrate preferences and rates of metabolism (for a review on the CYP P450 super family see [16]). Of the hepatic CYP P450 enzymes, CYP 2D6 is estimated to make up only four percent of the total human P450 liver content, yet it is estimated to metabolize 20-25% of commonly used drugs. Despite the importance of CYP 2D6 in drug metabolism, the enzyme is highly polymorphic in various human populations with more than 74 alleles reported to date [17]. CYP 2D6 phenotypes can be classified into four groups: poor metabolizers (PMs), intermediate metabolizers (IMs), extensive metabolizers (EMs), and ultra-rapid metabolizers (UMs). The frequency of PMs varies among populations and can have large interethnic differences with estimates in the range of 0-19% in African populations, 0–4.8% in Asian populations, and 1.5-8.9% in Caucasian populations (for a more detailed description of CYP 2D6 genotypes and PM frequencies see [18-20]). These genetic and phenotypic differences contribute to significant differences in the metabolism of CYP 2D6 substrates.

**Figure 1:**
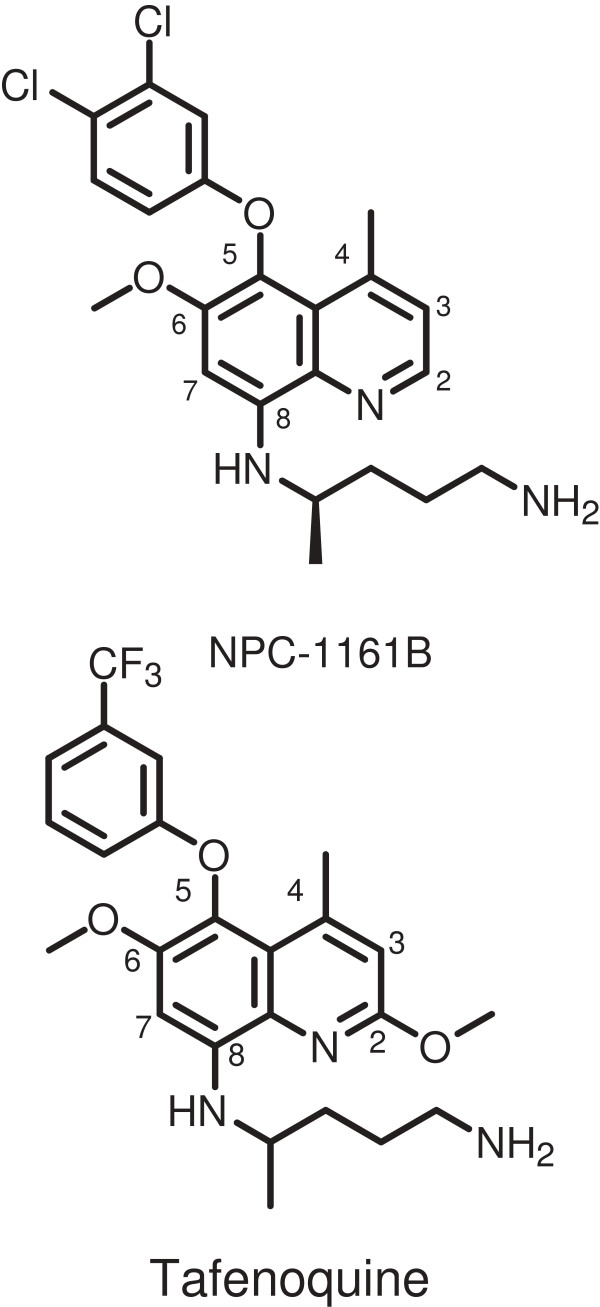
**8-aminoquinoline anti-malarial compounds NPC-1161B and tafenoquine.** Shown are the structures of the compounds utilized in this study. The quinoline rings are numbered (2–8) for reference.

Previous studies with PQ indicated that CYP 2D6 metabolism was required for anti-malarial activity [6] and it remained unclear if other 8AQ compounds similarly require CYP 2D6 activation. For this purpose, the 8AQ compounds NPC-1161B and tafenoquine (TQ) (Figure 1) were chosen and tested using CYP 2D knock-out and humanized/CYP 2D6 knock-in mice. The CYP 2D knock-out mice have a deletion of the mouse CYP 2D gene cluster and do not express a functional CYP 2D enzyme capable of metabolizing CYP 2D6 substrates [21]. The humanized/CYP 2D6 knock-in mice have the deletion of the mouse CYP 2D gene cluster, which is replaced with a human CYP2D6 expression cassette [21]. Both NPC-1161B and TQ are promising 8AQ molecules in development as TQ has been tested in several Phase II and III clinical studies [22-26] and is in late stage development as an anti-malarial prophylactic agent. NPC-1161B is a promising 8AQ in late preclinical development. Both compounds have an O-aryl substituent at the 5 position. In part, these compounds were selected to determine whether this key difference with respect to PQ might overcome/alter CYP 2D dependence for efficacy.

## Methods

### Chemicals used

NPC-1161B was supplied by Dr. Larry Walker and Dr. Dhammika Nanayakkara from the Center for Natural Products Research, University of Mississippi, and TQ was obtained from the Walter Reed Army Institute of Research chemical repository.

### *Plasmodium berghei* sporozoites isolation, inoculation and viability check

*Plasmodium berghei* sporozoites (luciferase expressing) were obtained from laboratory-reared female *Anopheles stephensi* and maintained at 18°C for 17 to 22 days after feeding on malaria-infected Swiss CD-1/ICR mice. Salivary glands were extracted from malaria-infected mosquitoes and sporozoites obtained. Briefly, mosquitoes were separated into abdomen and head/thorax. Heads and thoraxes were triturated with a mortar and pestle and suspended in medium RPMI 1640 containing 1% C57BL/6 mouse serum (Rockland Co, Gilbertsville, PA, USA). A total of 50–80 heads with glands were placed into a 0.5 ml Osaki tube on top of glass wool with enough dissection media to cover the heads. The Osaki tube was kept on ice until all mosquitoes had been dissected. Sporozoites isolated from the same batch of mosquitoes were inoculated into C57BL/6, 2D knock-out and 2D knock-out/2D6 knock-in C57BL/6 mice on the same day to control for biological variability in sporozoite preparations. Each mouse was inoculated intravenously in the tail vein with approximately 10,000 sporozoites suspended in 0.1 ml volume on day 0.

To ensure that inoculated sporozoites were viable following the isolation procedure, they were stained with a vital dye containing fluorescein diacetate (50 mg/ml in acetone) and ethidium bromide (20 μg/ml in phosphate-buffered saline; Sigma Chemical Co, St. Louis, MO, USA) and counted in a haemocytometer. The viability of sporozoites ranged from 90 to 100%.

### Animals

Male eight- to 12-week old C57BL/6, 2D knock-out and 2D knock-out/2D6 knock-in C57BL/6 mice (Taconic, Hudson, NY, USA) were used. On arrival, the animals were acclimated for seven days (quarantine). The animals were housed in a cage maintained in a room with a temperature range of 64-79°F, 34-68% relative humidity and a 12-hr light/dark cycles. Food and water were provided during quarantine and throughout the study. The animals were fed a standard rodent maintenance diet. All animal studies were performed under IACUC approved protocols. All animal use, care and handling was performed in accordance with the current Guide for the Care and Use of Laboratory Animals (1996).

### Test compounds and administration

Compounds tested in these experiments were dosed based on the body weight at the time of preparation of the suspension solution. The suspension solution of oral agents were prepared in 0.5% (w/v) hydroxyethyl cellulose and 0.2% (0.5% HECT, v/v) Tween-80 in distilled water, using homogenizer (PRO Scientific Inc, Monroe, CT, USA) with 10 mm open-slotted generator to homogenize drug powder mixture at 20,000-22,000 rpm for 5 min in ice bath. A once-a-day, three consecutive day-treatment regimen (−1, 0, 1 day) was used in all assessments. Drug suspensions were transferred to a 20-ml bottle, drawn into a 1-ml syringe, and delivered via intragastric feeder (18 gauge) to the designated recipient.

### *In vivo* imaging system spectrum

All *in vivo* imaging system (IVIS) methods utilized have been described previously [6]. Briefly NPC-1161B and TQ were administered orally on days −1, 0 and 1 with respect to sporozoite inoculation. At 24, 48 and 72 hr post-sporozoite infection, all inoculated mice were tested using the Xenogen IVIS-200 Spectrum (Caliper Life Sciences, Hopkinton, MA, USA) IVIS instrument. Additionally, blood-stage infections were measured by a flow cytometry system (FC500 MPL, Beckman Coulter, Miami, FL, USA). Positive and negative controls were used for the IVIS calibration in each test. D-Luciferin potassium salt, (Xenogen, California and Goldbio, St Louis, MO, USA), the luciferase substrate, was intraperitoneally inoculated into mice at a concentration of 200 mg/kg 15 min before luminescence analysis. Three min post-luciferin administration the mice were anesthetized with isoflurane. The mice were then positioned ventral side up in the IVIS on the 37°C platform. The mice continued to receive isoflurane through nose cone delivery. The camera exposure time was 5 min for the 24, 48 and 72 hr time points with f-stop = 1 and large binning setting. Photons emitted from specific regions were quantified using Living Image® 3.0 software.

## Results

### NPC-1161B and tafenoquine efficacy in CYP 2D knock-out mice

**Figure 2:**
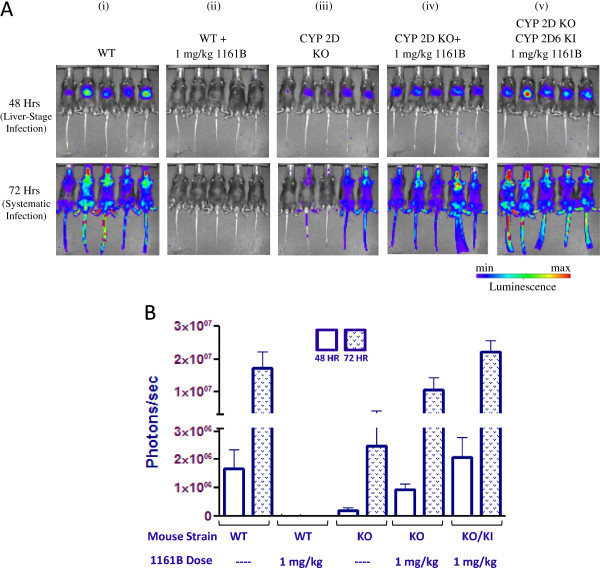
**Dissemination of malaria parasites in C57BL/6 wild-type (WT), CYP 2D knock-out, and humanized/CYP 2D6 knock-in mice 48 and 72 hr post-inoculation with luciferase expressing *Plasmodium berghei *in the absence or presence of NPC-1161B. A** (i-v). IVIS images of various mouse strains 48 and 72 hr post-inoculation. The mice of each group tested are shown and the corresponding luminescence signal indicated. **B**. Quantitated luminescence signal for each group tested 48 and 72 hr post-inoculation.

In order to determine if NPC-1161B (Figure 1) requires activation through CYP 2D metabolism, NPC-1161B was tested at its ED_100_ value (1 mg/kg) in C57BL/6 mice infected with luciferase expressing *P. berghei*. The IVIS luminescence results for NPC-1161B are shown in Figure 2A and quantitated luminescence values from parasite burden in Figure 2B. The 48-hr IVIS measurements indicate levels of *P. berghei* liver infection while the 72-hr measurements correspond to systemic *P. berghei* infection. Of the five wild type (WT) (C57BL/6) mice infected with sporozoites and not treated with NPC-1161B, all five exhibited robust luminescence signal as indicated by the IVIS images at 48 and 72 hr (panel i). The 1161B-treated WT group exhibited no luminescence signal at either 48 or 72 hr, indicating that NPC-1161B prevented *P. berghei* infection in WT mice (panel ii). Panel iii shows the results from CYP 2D knock-out mice infected with *P. berghei* as an infection control. Despite lower luminescence signals in this group, five of the five mice showed parasite burden/infection throughout the experiment. When NPC-1161B was tested at its ED_100_ in mice containing a deletion of the nine mouse CYP 2D genes (nearest mouse orthologue to human CYP 2D6), there was comparable luminescence signal to the WT control *P. berghei* infected mice at both 48 and 72 hr (panel iv) indicating that NPC-1161B was inactive in the CYP 2D knock-out mice. To investigate the effect of re-introducing a CYP 2D enzyme, NPC-1161B was tested at its ED_100_ in a humanized mouse strain where the CYP 2D gene cluster has been removed and replaced with the human CYP 2D6 gene and infected with *P. berghei* sporozoites (panel v). Of the five mice tested, all five mice had similar luminescence signals as compared to the WT control *P. berghei*-infected mice not treated with any compound. These results indicate that replacing the CYP 2D gene cluster with human CYP 2D6 was not sufficient to fully restore the activity of NPC-1161B.

**Figure 3:**
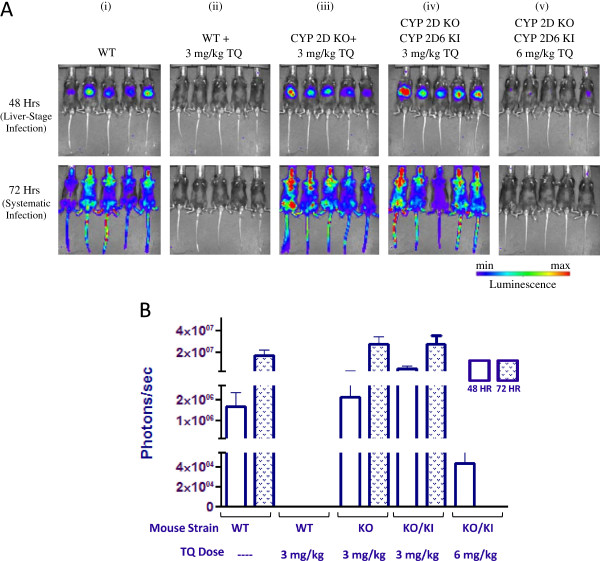
**Dissemination of malaria parasites in C57BL/6 wild-type (WT), CYP 2D knock-out, and humanized/CYP 2D6 knock-in mice 48 and 72 hr post-inoculation with luciferase expressing *Plasmodium berghei *in the absence or presence of tafenoquine. A** (i-v). IVIS images of various mouse strains 48 and 72 hr post-inoculation. The mice of each group tested are shown and the corresponding luminescence signal indicated. **B**. Quantitated luminescence signal for each group tested 48 and 72 hr post-inoculation.

Tafenoquine (Figure 1) was also tested in the CYP 2D knock-out mouse model. IVIS results are shown in Figure 3A and quantitated luminescence values from parasite burden in Figure 3B. Panel i shows the WT mice infected with *P. berghei* and panel ii shows the *P. berghei*-infected WT mice treated with TQ at its ED_100_ (3 mg/kg). There was no luminescence signal at either 48 or 72 hr post -infection in five out of the five mice treated, indicating that TQ prevented *P. berghei* infection. Panel iii shows *P. berghei-*infected CYP 2D knock-out mice treated with TQ at its ED_100_ (3 mg/kg). Each of the five mice showed robust luminescence indicating that TQ was not metabolized to the active form of the molecule required for anti-malarial activity when the CYP 2D gene cluster was removed. When TQ was tested in the humanized/CYP 2D6 knock-in mouse strain at its ED_100_, five out of the five mice showed robust luminescence (panel iv), indicating that replacing the CYP 2D gene cluster with human CYP 2D6 was not sufficient to fully restore the activity of TQ. To determine if this effect could be compensated for, TQ was tested at two times its ED_100_ in the humanized/CYP 2D6 knock-in mouse strain. The results in panel v show that there was less luminescence signal in all five mice at 48 hr (as compared to WT-infected controls) and no detectable luminescence signal at 72 hr post-inoculation.

The results in Figures 2 and 3 correspond to *P. berghei* infection 48 and 72 hr post-inoculation. In order to determine if the initial IVIS measurements translated to systemic infection, blood stage parasite burden measurements were made out to 31 days post-inoculation and the results shown in Table 1. The mouse strains tested along with the corresponding NPC-1161B/TQ doses given are indicated. The results for NPC-1161B (panel i) are consistent with those shown in Figure 2 in that five out of five WT mice treated with NPC-1161B never developed *P. berghei* infection. All other NPC-1161B groups were euthanized before day 22 due to blood stage parasite burden.

**Table 1:** **Cure rates in C57BL/6 wild-type (WT), CYP 2D knock-out, and humanized/CYP 2D6 knock-in mice 31 days post-inoculation with *Plasmodium berghei *** **in the absence or presence of NPC-1161B and tafenoquine**

**NCP-1161B**			
**i**	**Mouse strain**	**1161B Dose**	**Cure**
	**WT**	**-**	**0/5**
	**WT**	**ED**_**100**_	**5/5**
	**2DKO**	**-**	**0/5**
	**2DKO**	**ED**_**100**_	**0/5**
	**2DKO/2D6KI**	**ED**_**100**_	**0/5**
**Tafenoquine (TQ)**			
**ii**	**Mouse strain**	**TQ Dose**	**Cure**
	**WT**	**-**	**0/5**
	**WT**	**ED**_**100**_	**5/5**
	**2DKO**	**-**	**0/5**
	**2DKO**	**ED**_**100**_	**0/5**
	**2DKO/2D6KI**	**ED**_**100**_	**0/5**
	**2DKO/2D6KI**	**2 × ED**_**100**_	**4/5**

A cure is indicated if there was no detectable parasite burden in blood measurements after 31 days post-inoculation.

Tafenoquine blood parasite burden results are shown in Table 1 and are consistent with the IVIS results shown in Figure 3. Five out of five WT mice treated with TQ had no detectable parasite burden after 31 days, in comparison to the WT infection control or the CYP 2D knock-out mice treated with TQ. TQ was only active in the humanized/CYP 2D6 knock-in mice at two times its ED_100_. Four out of five mice were cured in the two times ED_100_ group. All other TQ groups were euthanized before day 22 due to blood parasite burden. These results along with those above for NPC-1161B illustrate that both TQ and NPC-1161B are active in the presence of the CYP 2D gene cluster. Activity was partially restored when the dose of TQ was adjusted to account for differences between mouse CYP 2D and human CYP 2D6 enzyme activity.

## Conclusions

Here direct preclinical evidence is presented that the 8AQ compounds NPC-1161B and TQ require metabolism by the CYP 2D enzyme class for activity. Human CYP 2D6 is involved in the metabolism of 20-25% of commonly used drugs on the market and is the most polymorphic of all CYP P450 enzymes [18,20]. These data demonstrate the critical role that CYP 2D enzymes play in the metabolic activation of NPC-1161B and TQ to the active form(s) of the molecules responsible for anti-malarial activity.

As might be expected, there appear to be differences between the mouse and human CYP 2D enzymes, as the anti-malarial activity of NPC-1161B and TQ were not restored in the CYP 2D knock-out/humanized/CYP 2D6 knock-in mice when tested at their respective ED_100_ (1 mg/kg and 3 mg/kg, respectively) values. In previous studies, the anti-malarial activity of PQ was partially restored when tested at its ED_100_ in the CYP 2D knock-out/humanized/CYP 2D6 knock-in mice [6]. While both NPC-1161B and TQ failed to achieve efficacy in CYP 2D knock-out/humanized/CYP 2D6 knock-in mice when tested at their corresponding ED_100_ values, anti-malarial activity was restored in the CYP 2D knock-out/humanized/CYP 2D6 knock-in experiments when the dose of TQ was doubled from its corresponding ED_100_ value (3 mg/kg to 6 mg/kg; NPC-1161B was not tested at two times its ED_100_ due to limited numbers of CYP 2D KO/CYP2D6 KI mice). Additionally, despite NPC-1161B and TQ belonging to the same structural class as PQ, there is little information available on the metabolism of these longer half-life molecules by CYP 2D6. It still remains uncertain if there are any other CYP enzymes capable of metabolising NPC-1161B and TQ, however, under the experimental conditions described above, the CYP 2D family is of primary importance for anti-malarial activity in mice and likely in humans. Further studies are required to understand the mechanism by which CYP 2D6 activates the NPC-1161B and TQ.

The lack of anti-malarial activity for TQ and NPC-1161B when tested in the CYP 2D6 knock-in mice could largely be attributed to intrinsic differences between the two CYP 2D enzymes (human *vs.* murine) as several reports have indicated differences in substrate metabolism and tissue expression between orthologues [21,27]. Scheer *et al*. showed using the CYP 2D6 reporter substrate bufuralol, only about a 53% recovery of bufuralol 1′-hydroxylase activity in microsomes generated from livers of the humanized CYP 2D6 knock-in animals versus those from the wild-type [21]. The CYP 2D6 knock-in mice have reduced enzymatic activity as compared to the WT CYP 2D mice that is required for 8AQ activation. Interestingly, this information could be utilized for the dose adjustment of 8AQs in humans that have reduced CYP 2D6 activity, as in some IM phenotypes as is done with other CYP 2D6-dependent drugs such as the anticancer drug tamoxifen [28]. This highlights the potential necessity of pharmacogenomic-guided therapy, with optimal dosing tailored for individual patients based on their CYP 2D6 genotype. It seems that the differences in mouse CYP 2D *vs.* human CYP 2D6 metabolism required for the anti-malarial activity of the 8AQs is more pronounced for the longer half-life 5-O-aryl analogues (TQ and NPC-1161B) as compared to PQ. TQ and NPC-1161B were not tested at two times their ED_100_ values in the CYP 2D knock-out mice because it is unlikely that any dose adjustment in mice lacking any functional CYP 2D enzyme(s) would result in restoration of activity as previously reported for PQ [6].

Taking the results presented above in context with literature reports, it is reasonable to conclude several things about NPC-1161B and TQ. 1). The anti-hypnozoite activity of NPC-1161B and TQ is dependent on CYP 2D activation. 2). Both NPC-1161B and TQ will likely fail for either causal prophylaxis and/or treatment indications in patients with CYP 2D6 genotypes resulting in the PM phenotype and may require dose modification in some patients with an IM phenotype. It is interesting to note that at the TQ and NPC-1161B doses tested in the knock-out experiments described above, no blood schizonticidal activity was observed. It is unclear as to how/if the previously reported slow intrinsic blood schizonticidal activity of TQ and NPC-1161B [3,29,30] is linked to CYP 2D6 metabolism. Further work is required in order to determine if CYP 2D6 metabolism is required for the blood schizonticidal activity of TQ and NPC-1161B in humans and whether this blood schizonticidal activity is sufficient to allow TQ and NPC-1161B to act as prophylactic agents in the absence of CYP 2D6 activation. Despite these issues, the requirement for CYP 2D6 activation for activity in the CYP 2D knock-out mouse model sheds light on another controversial issue that has surrounded the 8AQ class for decades: resistance.

There have been numerous reports in the literature of PQ failures that are associated with PQ resistance [1,31,32]. This “resistance” refers to the inability of PQ to clear the hypnozoite form of the *Plasmodium* parasite. There has been confusion around the idea of PQ resistance as there are many confounding factors associated with the various reports, such as patient population, patient adherence, dosing regimen, and concurrent blood schizonticidal therapy [1,33]. The requirement of CYP 2D6 activation for PQ activity is another factor that needs to be taken into consideration when reporting PQ resistance. Interestingly, the reported PQ failure rates seem to align with CYP 2D6 polymorphic allelic frequencies for the PM genotype [1] as individuals with this genotype will likely fail PQ therapy [15]. This is not a likely coincidence and calls into question the existence of PQ resistance and/or *Plasmodium* resistance to the 8AQ class in general, particularly since the results reported herein suggest that 8AQs likely have a similar mechanism(s) of anti-malarial activity which is mediated through CYP 2D6 activation. TQ and NPC-1161B do not have the wealth of clinical data that exists for PQ, so the exis**t**ence of TQ and NPC-1161B treatment failures is more difficult to interpret. Because TQ and NPC-1161B require CYP 2D activation for activity, rates of treatment/prophylactic failures would likely be in line with those noted for PQ use for both compounds when administered to humans. If insurmountable, this would present a major pharmacogenomic liability for the 8AQ class of anti-malarial compounds. New drugs with anti-hypnozoite activity are desperately needed to combat relapsing strains of malaria and future research and development efforts should ensure the complete dissociation between CYP 2D6 metabolism and anti-hypnozoite activity of new potential anti-malarial agents.

## Abbreviations

8AQ: 8-aminoquinoline; CYP: Cytochrome P450; PQ: Primaquine; TQ: Tafenoquine; G6PD: Glucose-6-phosphate dehydrogenase; IVIS: *in vivo* imaging system; WT: Wild type; ED100: 100% efficacious dose.

## Competing interests

The authors declare that they have no competing interests.

## Authors’ contributions

SRM, JCS, GAR drafted the manuscript, contributed to the experimental design, conducted data analyses, and assisted in study coordination. RJS, JWB, VZ, GD, MH, BS, VM, QL, LW, and LR contributed to the experimental design, experimental coordination, and assisted with drafting the manuscript. DC, QZ, NR, RM, and JC conducted all animal experiments and assisted with data analyses. LAW and NPDN assisted in compound synthesis and study design. BSP conceived of the study, and participated in its design and coordination. All authors read and approved the final manuscript.

## References

[B1] Baird JK Hoffman SL Primaquine therapy for malaria Clin Infect Dis 2004 39 1336 1345 10.1086/424663 15494911

[B2] Rieckmann KH McNamara JV Kass L Powell RD Gametocytocidal and sporontocidal effects of primaquine upon two strains of *Plasmodium falciparum* Mil Med 1969 134 802 819 4987059

[B3] Dow GS Gettayacamin M Hansukjariya P Imerbsin R Komcharoen S Sattabongkot J Kyle D Milhous W Cozens S Kenworthy D Miller A Veazey J Ohrt C Radical curative efficacy of tafenoquine combination regimens in *Plasmodium cynomolgi*-infected Rhesus monkeys (*Macaca mulatta*) Malar J 2011 10 212 10.1186/1475-2875-10-212 21801400 PMC3161915

[B4] Maude RJ Socheat D Nguon C Saroth P Dara P Li G Song J Yeung S Dondorp AM Day NP White NJ White LJ Optimising strategies for *Plasmodium falciparum* malaria elimination in Cambodia: primaquine, mass drug administration and artemisinin resistance PLoS One 2012 7 e37166 10.1371/journal.pone.0037166 22662135 PMC3360685

[B5] Jin X Pybus BS Marcsisin SR Logan T Luong TL Sousa J Matlock N Collazo V Asher C Carroll D Olmeda R Walker LA Kozar MP Melendez V An LC-MS based study of the metabolic profile of primaquine, an 8-aminoquinoline antiparasitic drug, with an in vitro primary human hepatocyte culture model Eur J Drug Metab Pharmacokinet 2013 [Epub ahead of print] doi: 10.1007/s13318-013-0139-8 10.1007/s13318-013-0139-8 23797843

[B6] Pybus BS Marcsisin SR Jin X Deye G Sousa JC Li Q Caridha D Zeng Q Reichard GA Ockenhouse C Bennett J Walker LA Ohrt C Melendez V The metabolism of primaquine to its active metabolite is dependent on CYP 2D6 Malar J 2013 12 212 10.1186/1475-2875-12-212 23782898 PMC3689079

[B7] Pybus BS Sousa JC Jin X Ferguson JA Christian RE Barnhart R Vuong C Sciotti RJ Reichard GA Kozar MP Walker LA Ohrt C Melendez V CYP450 phenotyping and accurate mass identification of metabolites of the 8-aminoquinoline, anti-malarial drug primaquine Malar J 2012 11 259 10.1186/1475-2875-11-259 22856549 PMC3438098

[B8] Constantino L Paixao P Moreira R Portela MJ Do Rosario VE Iley J Metabolism of primaquine by liver homogenate fractions. Evidence for monoamine oxidase and cytochrome P450 involvement in the oxidative deamination of primaquine to carboxyprimaquine Exp Toxicol Pathol 1999 51 299 303 10.1016/S0940-2993(99)80010-4 10445386

[B9] Ganesan S Tekwani BL Sahu R Tripathi LM Walker LA Cytochrome P(450)-dependent toxic effects of primaquine on human erythrocytes Toxicol Appl Pharmacol 2009 241 14 22 10.1016/j.taap.2009.07.012 19616568

[B10] Vasquez-Vivar J Augusto O Hydroxylated metabolites of the antimalarial drug primaquine. Oxidation and redox cycling J Biol Chem 1992 267 6848 6854 1313024

[B11] Augusto O Schreiber J Mason RP Direct ESR detection of a free radical intermediate during the peroxidase-catalyzed oxidation of the antimalarial drug primaquine Biochem Pharmacol 1988 37 2791 2797 10.1016/0006-2952(88)90042-1 2840077

[B12] Augusto O Weingrill CL Schreier S Amemiya H Hydroxyl radical formation as a result of the interaction between primaquine and reduced pyridine nucleotides. Catalysis by hemoglobin and microsomes Arch Biochem Biophys 1986 244 147 155 10.1016/0003-9861(86)90103-7 3004336

[B13] Morais Mda S Augusto O Peroxidation of the antimalarial drug primaquine: characterization of a benzidine-like metabolite with methaemoglobin-forming activity Xenobiotica 1993 23 133 139 10.3109/00498259309059369 8498077

[B14] Vasquez-Vivar J Augusto O Oxidative activity of primaquine metabolites on rat erythrocytes in vitro and in vivo Biochem Pharmacol 1994 47 309 316 10.1016/0006-2952(94)90022-1 8304975

[B15] Bennett JW Pybus BS Yadava A Tosh D Sousa JC McCarthy WF Deye G Melendez V Ockenhouse CF Primaquine failure and cytochrome P-450 2D6 in *Plasmodium vivax* malaria N Engl J Med 2013 369 1381 1382 10.1056/NEJMc1301936 24088113

[B16] Anzenbacher P Anzenbacherova E Cytochromes P450 and metabolism of xenobiotics Cell Mol Life Sci 2001 58 737 747 10.1007/PL00000897 11437235 PMC11337355

[B17] Zhou SF Polymorphism of human cytochrome P450 2D6 and its clinical significance: Part I Clin Pharmacokinet 2009 48 689 723 10.2165/11318030-000000000-00000 19817501

[B18] Bernard S Neville KA Nguyen AT Flockhart DA Interethnic differences in genetic polymorphisms of CYP2D6 in the U.S. population: clinical implications Oncologist 2006 11 126 135 10.1634/theoncologist.11-2-126 16476833

[B19] Bogni A Monshouwer M Moscone A Hidestrand M Ingelman-Sundberg M Hartung T Coecke S Substrate specific metabolism by polymorphic cytochrome P450 2D6 alleles Toxicol In Vitro 2005 19 621 629 10.1016/j.tiv.2005.04.001 15893449

[B20] Ingelman-Sundberg M Genetic polymorphisms of cytochrome P450 2D6 (CYP2D6): clinical consequences, evolutionary aspects and functional diversity Pharmacogenomics J 2005 5 6 13 10.1038/sj.tpj.6500285 15492763

[B21] Scheer N Kapelyukh Y McEwan J Beuger V Stanley LA Rode A Wolf CR Modeling human cytochrome P450 2D6 metabolism and drug-drug interaction by a novel panel of knockout and humanized mouse lines Mol Pharmacol 2012 81 63 72 10.1124/mol.111.075192 21989258

[B22] Walsh DS Wilairatana P Tang DB Heppner DG Jr Brewer TG Krudsood S Silachamroon U Phumratanaprapin W Siriyanonda D Looareesuwan S Randomized trial of 3-dose regimens of tafenoquine (WR238605) versus low-dose primaquine for preventing Plasmodium vivax malaria relapse Clin Infect Dis 2004 39 1095 1103 10.1086/424508 15486831

[B23] Walsh DS Looareesuwan S Wilairatana P Heppner DG Jr Tang DB Brewer TG Chokejindachai W Viriyavejakul P Kyle DE Milhous WK Schuster BG Horton J Braitman DJ Brueckner RP Randomized dose-ranging study of the safety and efficacy of WR 238605 (Tafenoquine) in the prevention of relapse of *Plasmodium vivax* malaria in Thailand J Infect Dis 1999 180 1282 1287 10.1086/315034 10479159

[B24] Walsh DS Eamsila C Sasiprapha T Sangkharomya S Khaewsathien P Supakalin P Tang DB Jarasrumgsichol P Cherdchu C Edstein MD Rieckmann KH Brewer TG Efficacy of monthly tafenoquine for prophylaxis of *Plasmodium vivax* and multidrug-resistant *P. falciparum* malaria J Infect Dis 2004 190 1456 1463 10.1086/424468 15378438

[B25] Hale BR Owusu-Agyei S Fryauff DJ Koram KA Adjuik M Oduro AR Prescott WR Baird JK Nkrumah F Ritchie TL Franke ED Binka FN Horton J Hoffman SL A randomized, double-blind, placebo-controlled, dose-ranging trial of tafenoquine for weekly prophylaxis against *Plasmodium falciparum* Clin Infect Dis 2003 36 541 549 10.1086/367542 12594633

[B26] Elmes NJ Nasveld PE Kitchener SJ Kocisko DA Edstein MD The efficacy and tolerability of three different regimens of tafenoquine versus primaquine for post-exposure prophylaxis of *Plasmodium vivax* malaria in the Southwest Pacific Trans R Soc Trop Med Hyg 2008 102 1095 1101 10.1016/j.trstmh.2008.04.024 18541280

[B27] Miksys SL Cheung C Gonzalez FJ Tyndale RF Human CYP2D6 and mouse CYP2Ds: organ distribution in a humanized mouse model Drug Metab Dispos 2005 33 1495 1502 10.1124/dmd.105.005488 16033950

[B28] Walko CM McLeod H Use of CYP2D6 genotyping in practice: tamoxifen dose adjustment Pharmacogenomics 2012 13 691 697 10.2217/pgs.12.27 22515611

[B29] Pradines B Mamfoumbi MM Tall A Sokhna C Koeck JL Fusai T Mosnier J Czarnecki E Spiegel A Trape JF Kombila M Rogier C In vitro activity of tafenoquine against the asexual blood stages of *Plasmodium falciparum* isolates from Gabon, Senegal, and Djibouti Antimicrob Agents Chemother 2006 50 3225 3226 10.1128/AAC.00777-06 16940138 PMC1563556

[B30] Delves M Plouffe D Scheurer C Meister S Wittlin S Winzeler EA Sinden RE Leroy D The activities of current antimalarial drugs on the life cycle stages of *Plasmodium*: a comparative study with human and rodent parasites PLoS Med 2012 9 e1001169 10.1371/journal.pmed.1001169 22363211 PMC3283556

[B31] Bright AT Alenazi T Shokoples S Tarning J Paganotti GM White NJ Houston S Winzeler EA Yanow SK Genetic analysis of primaquine tolerance in a patient with relapsing *vivax* malaria Emerg Infect Dis 2013 19 802 805 10.3201/eid1905.121852 23648098 PMC3647516

[B32] Langholz Kristensen K Dragsted UB Recurrent *Plasmodium vivax* malaria due to dose-dependent primaquine resistance: a case report Scand J Infect Dis 2013 46 63 65 23957539 10.3109/00365548.2013.822093

[B33] Fernando D Rodrigo C Rajapakse S Primaquine in *vivax* malaria: an update and review on management issues Malar J 2011 10 351 10.1186/1475-2875-10-351 22152065 PMC3306765

